# Standardization of *Alpinia calcarata* Roscoe rhizomes

**DOI:** 10.4103/0974-8490.72324

**Published:** 2010

**Authors:** L. S. R. Arambewela, L. D. A. M. Arawwawala

**Affiliations:** *Industrial Technology Institute, Herbal Technology Section, Bauddhaloka Mawatha, Colombo 7, Sri Lanka*

**Keywords:** *Alpinia calcarata*, phytochemicals, physico-chemical parameters

## Abstract

**Background:**

Rhizomes of *Alpinia calcarata* Roscoe (Family: Zingiberaceae) possess several bioactivities and are used in the traditional medicinal systems of Sri Lanka.

**Methods:**

The present investigation was carried out to standardize the rhizomes of *A. calcarata* by (a) screening for phytochemicals (b) determination of physico-chemical parameters and (c) development of a Densitogram.

**Results:**

Phytochemical screening revealed the presence of polyphenols, tannins, flavonoids, steroid glycosides and alkaloids in *A. calcarata* rhizomes. The percentages of moisture, total ash, acid insoluble ash, water soluble ash, ethanol extractable matter and water extractable matter were of 5.5 – 6.8, 8.3 – 8.8, 0.036 – 0.040, 7.2 – 7.8, 22.6 – 24.8 and 18.6 - 20.5 respectively.

**Conclusion:**

The results obtained from this study can be used to standardize rhizomes of *A. calcarata* grown in Sri Lanka.

## INTRODUCTION

The quality control of plant products is a general requirement to be fulfilled if a plant product is to be released to the market as a drug constituent. It is generally accepted that the plant kingdom offers tremendous potential in this field and should be exploited and used as supplement to the purely synthetic drugs on the market. However, since the synthetic drugs are subjected to severe quality control, the plant products also must comply with the same quality standards.[1] The secondary metabolites, such as alkaloids, flavonoids, lignins, terpenoids, steroids, glycosides, coumarins, and phenolic compounds, in plant materials produce the curative effect when they are used in the traditional medicinal practice. The composition of the secondary metabolites varies from plant species to species. Within the same species of plant, the composition of these compounds can vary with the nutrient composition of the soil, climatic season, developmental stage of the plant, natural association with other plants, storage of raw materials, and the types of processing methods, such as drying and extraction procedures.[2,3]

*Alpinia calcarata* Roscoe (Family: Zingiberaceae), is a rhizomatous perennial herb, which is commonly used in the traditional medicinal systems in Sri Lanka. The mature rhizomes are branched and dense with a light to dark brown color. The leaf of the plant is simple, alternative, 25–32 cm long and 2.5–5 cm broad.[4,5] *A. calcarata* is cultivated in tropical countries, including Sri Lanka, India, and Malaysia.[5] Experimentally, rhizomes of *A. calcarata* are shown to possess antibacterial,[6] antifungal,[7] anthelmintic,[8] antinociceptive,[9] antioxidant,[10] aphrodisiac,[11] gastroprotective,[12,13] and antidiabetic activities.[14] According to Arambewela and co-workers,[15] the major constituents in the essential oils of rhizome, root, and leaves of *A. calcarata* grown in Sri Lanka are different from that of Indian cultivars. Therefore, it is necessary to standardize *A. calcarata* rhizomes using quality control parameters to assure the quality of herbal preparations made from *A. calcarata* grown in Sri Lanka.

## MATERIALS AND METHODS

### Plant material

Fresh *A. calcarata* rhizomes were collected from home gardens in Western Province of Sri Lanka between the period of August and November. The plant material was identified and authenticated by the curator of National Herbarium, Royal Botanical Gardens, Peradeniya, Sri Lanka. A voucher specimen (AS 01) was deposited in the Industrial Technology Institute, Colombo 7, Sri Lanka.

### Preparation of the hot ethanolic extract

Fresh *A. calcarata* rhizomes were cut into small pieces and air dried for 12-14 days in the shade. Five hundred grams of powdered rhizomes were extracted with 1.5 L of ethanol using Soxhlet extraction apparatus for 4 h. The extraction was filtered and the filtrate was evaporated to dryness under reduced pressure at 50 °C (yield 18.5% w/w dry weight basis) and stored at 4 °C until use.

### Preparation of the hot water extract

Fresh *A. calcarata* rhizomes were cut into small pieces and air dried for 5–6 days in the shade. Five hundred grams of dried rhizomes were boiled with 2.5 L of distilled water for 4 h. The hot water extract was concentrated under vacuum at 60 °C and freeze-dried at –20 °C (yield 15.6% w/w dry weight basis) and stored at 4 °C until use.

### Preliminary phytochemical screening of *A. calcarata* extracts

The qualitative chemical tests were performed for both hot ethanolic extract (HEE) and hot water extract (HWE) according to the methods described by Farnsworth[16] with some modifications.

### Determination of presence/absence of polyphenolic compounds

Two to three drops of 1% FeCl_3_ solution was added to 2 mL portions (1%) of each extract. Phenolic compounds produce a deep violet color with ferric ions.

### Determination of presence/absence of tannins

The extract was diluted with water and added to diluted ferric chloride solution. Tannins give a blackish blue or green blackish color in the presence of ferric chloride.

### Determination of presence/absence of flavonoids

The extract was dissolved in methanol (50 %, 1–2 mL) by heating. Then metal magnesium and 5–6 drops of conc. hydrochloride acid (HCl) were added. The solution turns red when flavonoids are present.

### Determination of presence/absence of steroid glycosides

The extract was dissolved in equal volumes of acetic anhydride and CHCl_3_. The mixture was transferred to a dry test tube and conc. H_2_ SO_4_ acid was added at the bottom of the tube. Formation of a reddish brown or violet brown ring at the interface of the 2 liquids indicates the presence of steroids.

### Determination of presence/absence of alkaloids

The alkaloids were extracted by refluxing the sample with sufficient amount of water for about 2 h. The extract was concentrated on a rotor vapor, basified with NH_4_ OH and was extracted with CHCl_3_ (three times). Then the content was concentrated and 2 drops were spotted separately on a thin layer chromatography (TLC) plate. After development the plate was dried, Dragendorff’s reagent was sprayed onto them. Alkaloids give an orange color with Dragendorff’s reagent.

### Development of the densitograms for HEE and HWE

TLC fingerprints were developed for HEE and HWE using 15 μL of each extract (0.05 g/mL) and quantitatively analyzed by a densitometer (CS-9301PC, Shimadzu, Japan) at λ 254 nm.

Solvent systems for HEE: chloroform:hexane (1:1)

Solvent system for HWE: methanol:chloroform:cyclohexane:diethyl amine (0.5:2:1.5:1)

### Determination of physico-chemical parameters of *A. calcarata* rhizomes

Physicochemical parameters were determined for *A. calcarata* rhizomes according to methods described in WHO guidelines.^17^

### Determination of moisture content

The powdered material (1 g) was placed in a moisture dish and dried to a constant weight in an oven at 100–105 °C. The loss of weight (in mg/g) of air dried material was calculated as follows:

$$\%\mathrm{Moisture}\;\mathrm{content}\;=\;\frac{\mathrm{weight}\;\mathrm{loss}}{\mathrm{weight}\;\mathrm{of}\;\mathrm{sample}}\times 100$$

### Determination of total ash content

The powdered material (2 g) was accurately weighed and placed in a crucible. The material was spread in an even layer and it was ignited to a constant weight by gradually increasing the heat to 500–600 °C until it was white indicating the absence of carbon. The residual ash was allowed to cool in a dessicator. The content of total ash (in mg/g) of air-dried material was calculated as follows:

$$\%\mathrm{Total}\;\mathrm{ash}\;=\;\frac{\mathrm{Weight}\;\mathrm{of}\;\mathrm{ash}}{\mathrm{weight}\;\mathrm{of}\;\mathrm{sample}}\times 100$$

### Determination of acid insoluble ash content

HCl (2 N; 25 mL) was added to the crucible containing the total ash, covered with a watch glass, and boiled gently for 5 min. The watch glass was rinsed with 5 mL of hot water and the rinsed contents were added to the crucible. The acid insoluble matter was collected on an ashless filter paper and washed with hot water until the filtrate was neutral. The filter paper containing acid insoluble matter was transferred to the original crucible, dried on a hot plate, and ignited to a constant weight. The residue was allowed to cool in a dessicator and weighed. The content of the acid insoluble ash (in mg/g) of air-dried material was calculated as follows:

$$\%\;\mathrm{Acid}\;\mathrm{insoluble}\;\mathrm{ash}\;=\;\frac{\mathrm{weight}\;\mathrm{of}\;\mathrm{ash}}{\mathrm{weight}\;\mathrm{of}\;\mathrm{sample}}\times 100$$

### Determination of water soluble ash content

Water (25 mL) was added to the crucible containing the total ash, covered with a watch glass and boiled gently for 5 min. The watch glass was rinsed with 5 mL of hot water and added to the crucible. The water insoluble matter was collected on an ashless filter paper and washed with hot water. The filter paper containing the water insoluble matter was transferred to the original crucible, dried on a hot plate, and ignited to a constant weight. The water soluble ash content was calculated using the following equation.

$$\%\;\mathrm{Water}\;\mathrm{soluble}\;\mathrm{ash}\;=\;\frac{\mathrm{total}\;\mathrm{ash}\;\mathrm{content}\;-\;\mathrm{water}\;\mathrm{insoluble}\;\mathrm{residue}\;\mathrm{in}\;\mathrm{the}\;\mathrm{total}\;\mathrm{ash}}{\mathrm{weight}\;\mathrm{of}\;\mathrm{sample}}\times 100$$

### Determination of ethanol extractable matter

Accurately weighed powdered material (4 g) was placed in a glass stoppered conical flask. Ethanol (95%; 100 mL) was added to the flask and it was weighed to obtain the total weight, including the flask. Then, the flask was shaken well and allowed to stand for 1 h. A reflux condenser was attached to the flask and boiled gently for 1 h, and then it was cooled and weighed. The weight was readjusted to the original total weight by adding required amount of 95% ethanol. The flask was shaken well and filtered rapidly through a dry filter paper. After that, 25 mL of the filtrate was transferred to a tared flat bottomed dish and evaporated to dryness on a water bath. Then the dish was dried at 105 °C for 6 h and cooled in a dessicator and weighed. The content of extractable matter (in mg/g) air-dried material was calculated as follows:

$$\%\mathrm{Ethonal}\;\mathrm{extractable}\;\mathrm{matter}\;=\;\frac{\mathrm{weight}\;\mathrm{of}\;\mathrm{residue}}{\mathrm{weight}\;\mathrm{of}\;\mathrm{sample}}\times 4\times 100$$

### Determination of water extractable matter

The same procedure as described for the ethanol extractable matter was followed for the determination of water extractable matter using distilled water instead of 95% ethanol.

## RESULTS

### Test for polyphenolic compounds

A deep violet color appeared indicating the presence of phenolic compounds.

### Test for tannins

A green blackish color appeared revealing the presence of tannins.

### Test for flavonoids

An orange color with ferric chloride revealed the presence of flavonoids.

### Test for steroid glycosides

A violet brown ring formed at the interface of the 2 liquids, indicated the presence of steroids.

### Test for alkaloids

An orange color with Dragendorff’s reagent indicated the presence of alkaloids.

### Test for physicochemical parameters

Results are listed inTable 1.

**Table 1:** Physico-chemical parameters of *Alpinia calcarata* rhizomes

Physico-chemical parameters	% Amount (dry weight basis)
Moisture content	5.5–6.8
Total ash	8.3–8.8
Acid insoluble ash	0.036–0.040
Water soluble ash	7.2–7.8
Ethanol extractable matter	22.6–24.8
Water extractable matter	18.6–20.5

### The densitograms for HEE and HWE

**Figure 1:**
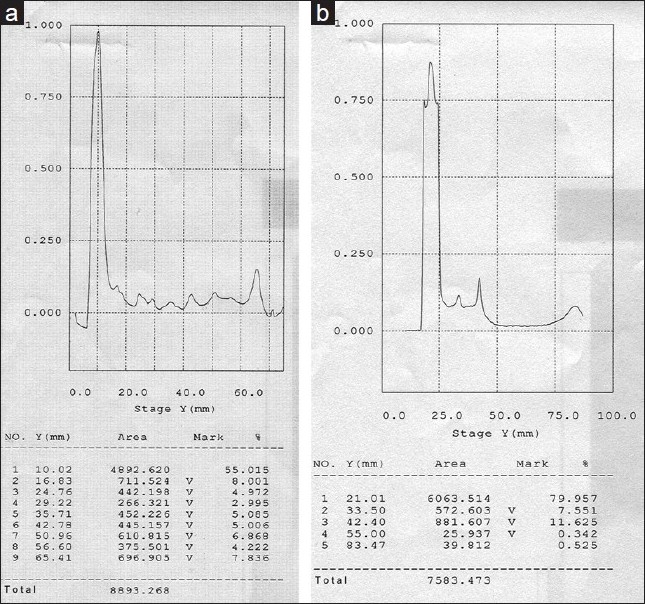
The densitograms for (a) hot ethanolic extract (HEE) and (b) hot water extract (HWE) at 254 nm

The densitograms of HEE and HWE [Figure 1] revealed the differences in the chemical constituents in these extracts.

## DISCUSSION

The quantitative determination of some pharmacognostic parameters is useful for setting standards for crude drugs. Phytochemical screening revealed the presence of polyphenolic compounds, tannins, flavonoids, steroid glycosides, and alkaloids in both HEE and HWE. Although the classes of phytochemicals present are common to both extracts, chemical constituents belonging to HEE differ from that of HWE. This is clearly evident from the densitograms obtained with these extracts. The physicochemical analysis of plant drugs is an important for detecting adulteration or improper handling of drugs. The total ash is particularly important in the evaluation of purity and quality of drugs. The ash value was determined by 3 different methods, which measured total ash, acid insoluble ash, and water soluble ash. The total ash method is employed to measure the total amount of material remaining after ignition.[18] The total ash usually consists of carbonates, phosphates, silicates, and silica, which include both physiologic ash and nonphysiologic ash. A high ash value is indicative of contamination, substitution, adulteration, or carelessness in preparing the crude drug for marketing.[19] Acid insoluble ash indicates contamination with silica, for example, earth and sand. Comparison of this with the total ash value of the same sample will differentiate between contaminating materials and variations of the natural ash of the drug. Water soluble ash is that part of the total ash content, which is soluble in water. It is a good indicator of the water soluble salts in the drug.[19]

Extractive values are representative of the presence of the polar or nonpolar extractable compounds in a plant material. The water soluble extractive of ginger is expected to be in the range of the 10% with respect to air dried material, and lowering of this extractive value indicates the addition of exhausted material with the original drug. The water soluble extractive value can be used to indicate poor quality, adulteration with any unwanted material, or incorrect processing of the crude drug during the process of drying, storage and so on.[19] Moisture is an inevitable component of crude drugs, which must be eliminated as far as practicable. Insufficient drying leads to spoilage by molds and bacteria and makes possible the enzymatic destruction of active principles.[17,19]

In conclusion, the results obtained from phytochemical screening studies and physico-chemical parameters can be used to standardize rhizomes of *A. calcarata* grown in Sri Lanka.
